# Collaborating toward improving food security in Nunavut

**DOI:** 10.3402/ijch.v72i0.21201

**Published:** 2013-08-05

**Authors:** Jennifer Wakegijig, Geraldine Osborne, Sara Statham, Michelle Doucette Issaluk

**Affiliations:** Government of Nunavut Department of Health, Iqaluit, Nunavut, Canada

**Keywords:** food security, strategy, Nunavut Food Security Coalition, Inuit, Nunavut

## Abstract

**Background:**

Community members, Aboriginal organizations, public servants and academics have long been describing a desperate situation of food insecurity in the Eastern Canadian Arctic.

**Objective:**

The Nunavut Food Security Coalition, a partnership of Inuit Organizations and the Government of Nunavut, is collaborating to develop a territorial food security strategy to address pervasive food insecurity in the context of poverty reduction.

**Design:**

The Nunavut Food Security Coalition has carried out this work using a community consultation model. The research was collected through community visits, stakeholder consultation and member checking at the Nunavut Food Security Symposium.

**Results:**

In this paper, we describe a continuous course of action, based on community engagement and collective action, that has led to sustained political interest in and public mobilization around the issue of food insecurity in Nunavut.

**Conclusions:**

The process described in this article is a unique collaboration between multiple organizations that has led to the development of a sustainable partnership that will inform policy development while representing the voice of Nunavummiut.

## Introduction

Food insecurity occurs when food systems are stressed so that food is not accessible, available and/or of sufficient quality (1). In Nunavut, 70% of Inuit pre-schoolers live in food insecure homes (2), and Nunavut students are more likely than other Canadian students to go to bed hungry because there is not enough food at home (3). Community-based studies indicate rates of food insecurity, whether it is children or adults, ranging from 50 to 80% (2,4–6). Considerable government and academic investment has been made to understand the determinants of and potential solutions to food insecurity in Nunavut. Many food security determinants, identified in the peer-reviewed literature, can largely be attributed to rapidly changing environmental and socio-economic conditions throughout the Arctic (Table I).

**Table I:** Determinants of food (in) security in the Eastern Arctic

		Determinants of food (in) security	

		Country food	Store food
Availability	Environmental	Altered migration patterns of wildlife Varied distribution of wildlife Seasonal disparity in wildlife	Inclement weather causing flight delays Changing sea ice dynamics causing sea-lift delays
	Socio-economic	Growing populations putting localized pressure on wildlife Lack of hunter in the household	Stores ordering enough supply to meet demand Lack of worker in the household Presence of community food programs (i.e. food bank, soup kitchen) Presence of government food programs (i.e. Breakfast Program)
Accessibility	Environmental	Shorter sea ice season preventing hunters from using the sea ice Longer open water season allowing hunters to go boating Unpredictable weather patterns More frequent storms Stronger and more variable winds causing white-out conditions	Isolation of communities Heavy reliance on external transportation networks Extreme weather events preventing people from leaving their homes
	Socio-economic	Level of traditional knowledge required to hunt/harvest wildlife Time needed to hunt/harvest High cost of hunting (i.e. equipment, gas) Weakening of sharing networks Gambling/substance abuse/addiction	High cost of food Insufficient financial resources required to purchase food Policies/regulations imposed from other jurisdictions that are not an appropriate fit in Nunavut Weakening social networks Gambling/substance abuse/addiction
Quality	Environmental	Contaminants affecting health of wildlife Freeze–thaw cycles preventing animals from adequately foraging	Flight delays causing spoilage
	Socio-economic	Traditional knowledge required to harvest the healthiest animals	Nutritional knowledge required to make healthy food choices Language barriers (i.e. English food labels hinder unilingual Inuit)
Use	Environmental	Changing environmental conditions leading to spoilage (i.e. caching)	N/A
	Socio-economic	Traditional knowledge required to prepare wildlife	Cooking skills required to prepare groceries Language barriers (i.e. English recipes hinder unilingual Inuit)

Presently, the Nunavut Food Security Coalition is engaged in a process to create a collaborative strategy, the Nunavut Food Security Strategy, which will identify commitments and objectives for all partners working towards the reduction of food insecurity in Nunavut. This paper provides a case study of the confluence of variables that have led to meaningful intersectoral movement on a significant public health issue in a vulnerable population. It highlights the importance of strategic planning and advocacy, and of seizing opportunities for action when political will, meaningful research results and public mobilization raise the profile of an issue. Specifically, this paper describes the political landscape and the process underway to develop a Nunavut Food Security Strategy, and it highlights the various milestones and actions that have culminated in a collaborative process which engages stakeholders from government, academia, civil society and multiple non-governmental organizations (NGOs).

## Food security in Nunavut

Food insecurity is entrenched in Nunavut by a myriad of factors (Table I). Changing environmental conditions have been widely recognized as impacting food security. For example, the availability of country food is compromised when animal migration routes are altered due to climate change, with some impacts already observable in some Eastern Arctic communities (7,8). Other environmental changes such as thinner ice, later ice freeze-up, earlier ice break-up, more variable snowfall, unpredictable weather, warmer temperatures, as well as more frequent and intense storms can impact the ability of Inuit hunters to access wildlife (7–10). Increasingly common extreme weather hazards, including high winds and blizzards, can delay shipments to communities, which are accessible only by air except during the ice-free season, and limit the availability of fresh food stocks in local stores (7,11,12).

Shifting socio-economic conditions have also been identified as a significant determinant of food security. With regards to the socio-cultural context, Inuit food security is negatively impacted by a reduction in the number of active hunters harvesting traditional food (7,11–13). The high cost of obtaining food is a prevalent food security determinant amongst Inuit throughout the circumpolar north (5,7,11–18). Compounding the issue of high costs and low incomes is concerns over money management skills (11,12), which have been attributed to the relatively recent shift to a cash-based economy among Inuit, as well as limited experience of western concepts of budgeting (19).

Four main components of food security are recognized by international agencies and academics as the primary social and environmental factors relevant to any food system: *availability* (sufficient quantities available consistently), *accessibility* (enough resources to obtain food), *quality* (adequate nutritional and cultural value) and *use* (required knowledge of how to utilize food) (5,7,20,21). It is suggested that for food security to exist, these various dimensions must be fulfilled. The following chart describes the components of food security as well as specific determinants relevant to Inuit in Canada.

It is evident that the determinants of food insecurity in Nunavut are wide-ranging. As such, the solutions to food insecurity must be as well. It is widely acknowledged that addressing this critical and complex issue is broader than the mandate of any one organization. Therefore, an integrated, coordinated, and collaborative approach involving commitment and resources from many partners is the only way to achieve meaningful impact on this profound issue affecting most residents of Nunavut. Further on in this paper we will describe how Nunavut's approach to improving food security is embedded in a larger mobilization towards poverty reduction and how this work is being coordinated through partnerships and intersectoral action. The authors intend to describe in detail how intersectoral action is currently occurring through the development and operation of the Nunavut Food Security Coalition.

## Strategic work and milestone events: looking toward a territorial food security strategy

**Fig. 1:**
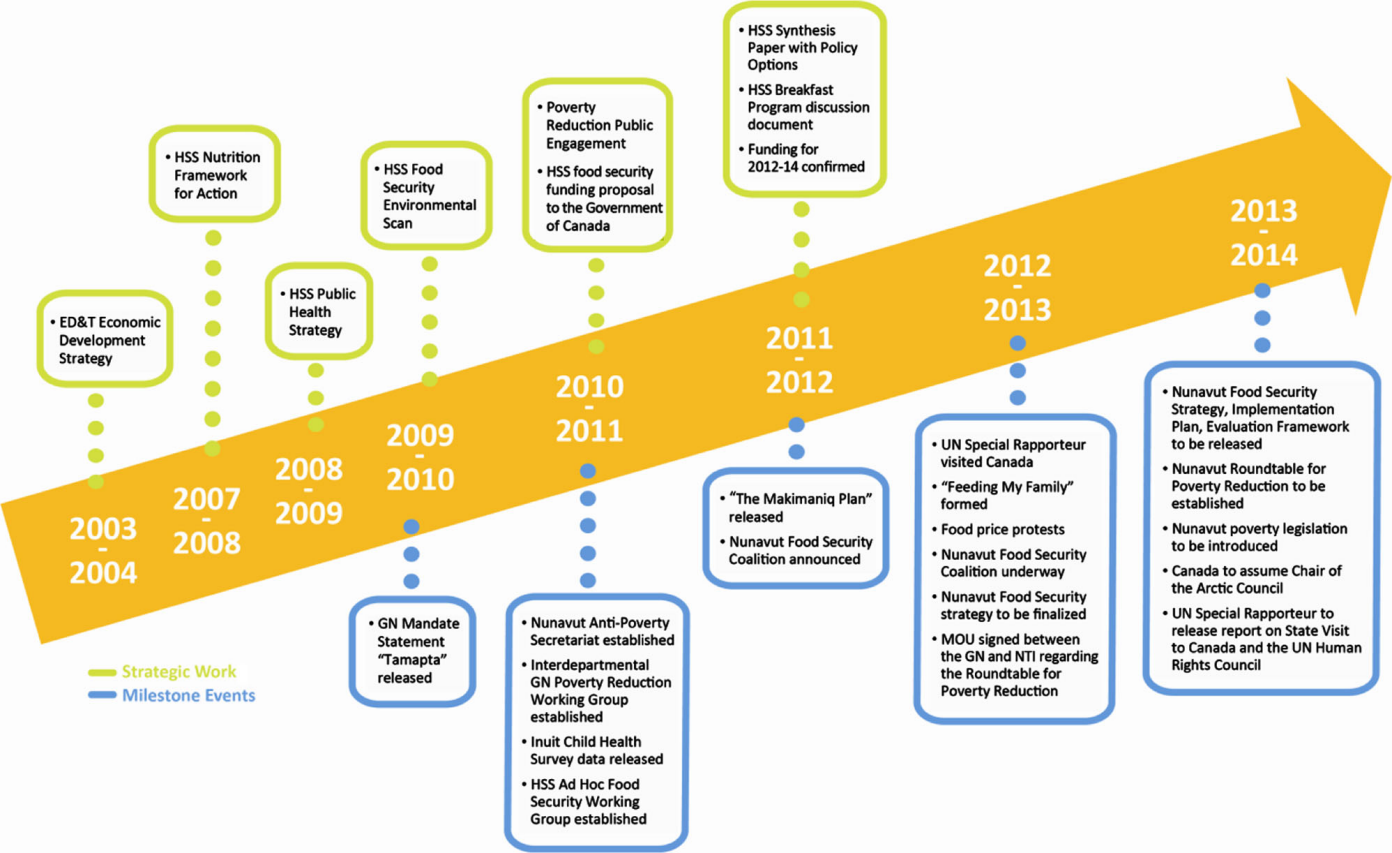
Timeline of the broad factors that helped to prioritize food security. *Note on April 1, 2013 the Department of Health and Social Services (HSS) became the Department of Health and a new Department of Family Services was formed.

Food security has become a political and public priority in Nunavut. Figure 1 identifies the chronological cascade of milestone events and coordinated strategic work, which has contributed to the central positioning of food security for intersectoral action. Events and work of major importance include: the release of compelling data on the state of food insecurity in Nunavut, unification of political will both at the territorial and federal level, mobilization of community members and identification of funds. Government, NGOs and civil society are collectively mobilizing and this promising process may yield the kind of collaboration that is needed to affect this issue. Further, the collective ownership of this work is likely to result in sustained action on this issue, both within and external to Nunavut.

### Milestone events

While food insecurity has been viewed as an important health priority by the Government of Nunavut, since its inception, many milestone events including those outlined in Fig. 1, have led to this issue becoming a leading priority for the Government of Nunavut, Nunavut Inuit Organizations and other Nunavut stakeholders.

#### Tamapta

In 2009, the Government of Nunavut (GN) released a statement of priorities, *Tamapta: Building Our Future Together 2009–2013*, emphasizing the importance of meeting the basic needs of Nunavummiut including housing, education, communication and recreation, amongst others (22). *Tamapta* states that “affordable, healthy food” should be ensured.

#### The Nunavut Anti-Poverty Secretariat

The Nunavut Anti-Poverty Secretariat was established on 1 April, 2010 to prepare and implement a poverty reduction strategy for Nunavut. The Secretariat is also responsible for the design and delivery of specific government programs to address poverty reduction, and those currently focus on country food distribution systems (23). The Nunavut Anti-Poverty Secretariat was previously housed within the GN's Department of Economic Development and Transportation (ED&T) and has moved to the new Department of Family Services as of 1 April, 2013. The Nunavut Anti-Poverty Secretariat works in partnership with Nunavut Tunngavik Incorporated (NTI). Together, these partners support the Nunavut Roundtable for Poverty Reduction, which is the organization responsible for the extensive public engagement process conducted in 2011, which was used to inform the territorial poverty reduction plan.

#### The Inuit Child Health Survey

In spring 2010, data were released from the Inuit Child Health Survey 2007–2008. This survey was a cross-sectional survey of the health status of 388 randomly selected Inuit children, aged 3–5 years, in 16 Nunavut communities, during the period 2007–2008. These findings confirmed, with greater statistical strength, what national surveys have implied for years, that is, that rates of food insecurity in Nunavut are considerably higher than in any other Canadian jurisdiction, and that food insecurity can be considered a crisis in Nunavut.

#### Department of Health Ad Hoc Food Security Working Group

In May of 2010, when the Inuit Child Health Survey was released, the Chief Medical Officer of Health (CMOH) and Territorial Nutritionist of the Department of Health and Social Services (HSS) convened an ad hoc working group within the department, to explore what advocacy could be done to raise the profile of this issue, and which policy initiatives could be undertaken (based on the environmental scan completed in 2009) to have a meaningful impact.

#### The Makimaniq Plan/Nunavut Food Security Coalition announcement

On 30 November, 2011, *The Makimaniq Plan: A shared approach to poverty reduction* was announced at the conclusion of Nunavut's Poverty Summit. The extensive public engagement process used to inform the document involved roundtable discussions held in each of Nunavut's 25 communities, followed by regional meetings in five communities, a policy forum and a poverty reduction summit. Six interrelated themes emerged as areas for action to reduce poverty in Nunavut, including:

- Collaboration and Community Participation
- Healing and Wellbeing
- Education and Skills Development
- Food Security
- Housing and Income Support
- Community and Economic Development

Under the theme of Food Security, one of the goals is “the establishment of a ‘Nunavut Food Security Coalition’” that would convene stakeholders from government, Inuit organizations, NGOs, business, and research to “develop a long-term, on-going, inclusive, and sustainable approach to food security in Nunavut” (24, p. 6).

#### UN Special Rapporteur visit to Canada

In May 2012, the issue of food insecurity in Canada was brought to light internationally by the state visit of the United Nations Special Rapporteur on the Right to Food, Dr. Olivier De Schutter. This event marked the first United Nations investigation of food security in a developed country. The purpose of the mission was to examine the way in which the right to food is being realized in Canada. The Government of Nunavut's Territorial Nutritionist made a presentation to the Special Rapporteur highlighting the severity of the issue in Nunavut, current initiatives and gaps, and the need for intersectoral collaboration.

#### “Feeding My Family” established

Subsequently, there has been major public mobilization around the issue of food insecurity in Nunavut. This momentum has been largely propelled by the creation of the “Feeding My Family” (FMF) group on the popular social media networking website, Facebook. FMF was created by a mother, who wanted to raise awareness about the fact that she, like many other people, struggled to feed her family. Since its creation in May 2012, over 20,000 people have joined FMF, which provides an online forum for discussion of food prices in the north.

#### Food price protests

Several protests against the high food prices in Nunavut have been staged throughout Canada, from large southern cities (i.e. Ottawa) to small northern communities (i.e. Grise Fiord). These protests are deemed unexpected yet momentous, as “Protesting is not something Inuit traditionally do. Inuit are taught [to] not cause disruption to others” (25).

#### Nunavut Food Security Coalition underway

Announced in November 2011, the Nunavut Food Security Coalition was formally established in June of 2012. The Coalition currently consists of representatives From seven Government of Nunavut departments and four Inuit organizations whose goal is to engage a broader group of partner organizations to create a collaborative strategy of programs, policies, and initiatives that are most likely to have a great impact on the food security of Nunavummiut. The focus is on identifying initiatives that can be undertaken within Nunavut, using existing resources.

### Strategic work

As outlined in Fig. 1, a number of strategic activities have coincided with the milestone events highlighted above. Each of these strategic activities has involved either the engagement of the public in identifying solutions, or synthesizing available academic information to formulate policy options, and throughout, partnerships have been building.

#### ED&T Economic Development Strategy

The 2003 *Nunavut Economic Development Strategy* was prepared by a broad coalition of government, Inuit organizations, as well as non-governmental and private sector groups that “[shared] a common desire to see Nunavut build a solid foundation for economic development and growth” (26). This strategy recommended that the territory explore how Nunavummiut could improve their diet and nutrition, how communities could strengthen local food production and distribution, and how reliance on southern food imports could be reduced through the establishment of a *Nunavut Commission on Food Autonomy* (26).

#### Nutrition in Nunavut – A Framework for Action

In 2007, the Department of Health developed a nutrition strategy entitled Nutrition in Nunavut - A Framework for Action which outlines priority nutrition issues, as well as goals and objectives that would support improved nutritional health (27). Food security was highlighted amongst the seven priority nutrition issues in the document, which include:

- Health Conditions
- Healthy Eating and Food Security
- Policies & Standards to Support Food Security & Healthy Eating
- Access to Care & Treatment for Nutrition-Related Health Concerns
- Nutrition-Focused Human Resources
- Inuit Employment Plan
- Financial Resources and Leadership

The Framework for Action involves 10 goals, each with relevant objectives. Goals supporting food security include providing a “range of nutrition services,” ensuring “sufficient nutritious food,” and supporting the “development of evidence-based policies and approaches” (27, p. 16, 18, 19).

#### Developing Healthy Communities: A Public Health Strategy for Nunavut

The Department of Health also created a Public Health Strategy entitled Developing Healthy Communities: A Public Health Strategy for Nunavut (28). Under its first priority of “Healthy Children and Families,” its third goal is “to improve food security for all families especially families with infants and children” (28, p. 8). The document recognizes that good nutrition plays a critical role in supporting lifelong well-being and disease prevention. It also acknowledges that Nunavummiut experience varying levels of hunger and malnutrition, and that the rates of associated diseases, such as diabetes, cancers, high blood pressure, rickets and anaemia are rising.

#### Food security environmental scan

With many government documents supporting action on food security (27–29), the Department of Health carried out an environmental scan in order to identify how to best address this issue. The document, entitled *Taking Action on Food Security in Nunavut: Current Initiatives and Opportunities*, highlighted current initiatives supporting food security in Nunavut undertaken by the territorial and federal government, non-governmental organizations and researchers (22). The report also identified opportunities to take advantage of such initiatives.

#### Poverty reduction public engagement

Seeking a collaborative approach for the preparation of a poverty reduction action plan, the Nunavut Roundtable for Poverty Reduction adopted a public engagement methodology (30). Nunavut’s public engagement for poverty reduction process was launched on 18 October, 2010, and preceded through three stages: dialogue, deliberation and decision making. An important goal of the Roundtable's public engagement process was the effort to learn about poverty in Nunavut from Nunavummiut that were experiencing it. As the community dialogues progressed during the first stage, what emerged was surprising uniformity in the views about what needs to be done to reduce poverty (30). This extensive public engagement process was initially intended to broadly inform *The Makimaniq Plan: A Shared Approach to Poverty Reduction*. However, it has also served to better understand the experience of food insecurity among Nunavummiut; many recommendations were made to improve existing policies, programs and initiatives. This process also resulted in suggestions as to how food security might be enhanced in the future. A comprehensive list of options for action has been compiled and is being considered by the Coalition throughout the strategy development process as part of its public engagement component.

#### Department of Health Synthesis Paper with Policy Options

Another important step in the process toward developing a territorial food security strategy was the creation of the Synthesis Paper with Policy Options (31). This document involved an extensive review and analysis of over 380 sources, including peer-reviewed literature and government documents, related to food security in Nunavut and other northern jurisdictions. Various policy options were identified and then reviewed through the lens of Nunavut priorities that were identified from government documentation, including *Tamapta*. The analysis of potential policy options prioritized initiatives that may have the potential to address multiple territorial priorities and goals in the areas of health, local economic development and employment, as well as promotion of cultural values. From this process, eight broad strategies emerged, under which 33 policy initiatives were classified. The eight food security categories include:

- Increase access to and use of country foods in daycares, schools and facilities
- Work with partners to explore targeted “commercialization” of country foods
- Promote and support informal country food networks
- Support active and young hunters
- Target infant and child feeding and feeding practices
- Community wide nutrition interventions
- Improve food affordability in communities
- Protect country food species

Similar to the list of options for action that resulted from the poverty reduction public engagement process, the list of policy options that resulted from this synthesis document is being considered by the Coalition throughout the strategy development process.

## The Nunavut Food Security Strategy development process

In June 2012, the Nunavut Food Security Coalition was established, consisting of seven Government of Nunavut departments and four Inuit organizations. The Coalition is led by Nunavut Tunngavik Incorporated, the Nunavut Anti-Poverty Secretariat and the Department of Health. Other partners in the coalition include: Kitikmeot Inuit Association; Kivalliq Inuit Association; Qikiqtani Inuit Association; Department of Culture and Heritage; Department of Education; Department of Executive and Intergovernmental Affairs; Department of Environment and the Nunavut Housing Corporation. In this partnership, the responsibility for the creation of a coalition and strategy lie with the Anti-Poverty Secretariat and NTI, while the Department of Health is contributing funding and technical leadership to the process.

**Fig. 2:**
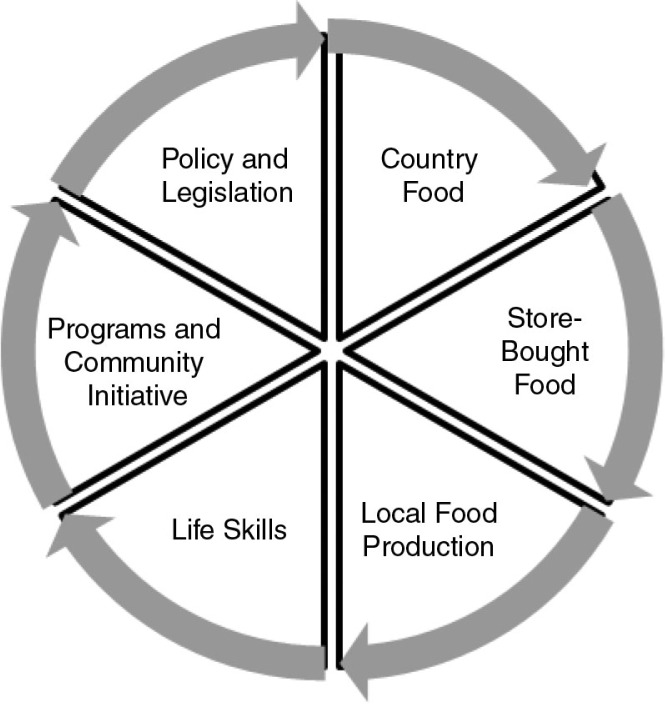
The Nunavut Food Security Coalition's 6 themes of food security.

The Coalition has identified six key themes around which the territorial food security strategy will be structured (Fig. 2). To better understand these themes, thematic discussions took place during fall 2012. These discussions engaged a broad group of partners to determine which policies, programs, and initiatives are most likely to have an impact on the food security of Nunavummiut.

Insights garnered from the thematic discussions culminated at the Nunavut Food Security Symposium, which was held January 22–24, 2013 in Iqaluit, Nunavut. The Symposium brought together 135 people who represented government departments, Inuit associations, NGOs, retailers, Hunters and Trappers Organizations, community-based organizations, and academic institutions. Panel discussions, presentations, and working sessions allowed participants to highlight existing initiatives that enhance food security, to explore the viability of other efforts to improve food security, and to identify knowledge gaps that should be filled in order to make responsible strategy decisions. After three days of productive dialogue, Nunavut-based partners emerged with priority areas for action on each theme. These priorities have been used as the basis to inform the Nunavut Food Security Strategy and Action Plan, which will be announced in spring 2013.

## Next steps and longevity of the strategy

While implementation of the Nunavut Food Security Strategy has yet to take place, it appears that there is considerable commitment on the part of many influential partners to support long-term implementation of the Strategy. As demonstrated in Fig. 1, a number of events that are already on the radar in 2013–2014, promise sustained action on this issue. The public nature of the strategy requires transparency and mutual accountability by the partners that have committed to it; as well the Nunavut Roundtable for Poverty Reduction and new anti-poverty legislation (Bill 59, Collaboration for Poverty Reduction Act) may well entrench action on food security into the future by key players within Nunavut. In addition, the UN Special Rapporteur on the Right to Food released a report on his country visit to Canada to the UN Human Rights Commission in December 2012. Furthermore, with Canada assuming the Chair of the Arctic Council in May 2013, and food security identified as a priority issue by this influential international body of Arctic Nations, it is not unreasonable to anticipate that public and political interest in this issue will remain high, and action on the issue will be sustained.

## Conclusion

Evidence-based policy making is challenging policymakers in population and public health to balance the pressures of time, political, economic, social, ethical and health ramifications of decision making (32). Many researchers and policy experts struggle with the challenge of either having inadequate evidence to support decision making, or with having ample evidence related to an issue and its solutions, but a lack of resources or political will to address them. This case study tells the tale of a convergence of positive government/non-government/research partnerships, timely release of data, strategic planning and positioning of an issue, public engagement and simultaneous external events that helped to secure action. It is hoped that this paper may provide motivation to researchers and advocates for socio-economic issues, as well as to public servants, and decision-makers to continue to build partnerships and alliances, and build evidence-based cases for action on issues of importance to society, so that when opportunities arise and the time is right, the elements are in place that will support advancement on the issue. In the case of food insecurity in Nunavut, a combination of strategic thinking and planning, a changing political climate and timely release of pertinent data has led to considerable intersectoral movement to address a critical socio-economic issue (food insecurity) in a population significantly challenged by this issue.
